# Delayed Recovery From Anesthesia Revealing Unrecognized Metabolic Acidosis and Hypokalemia in a Patient With Urinary Diversion

**DOI:** 10.7759/cureus.108530

**Published:** 2026-05-08

**Authors:** Priya Rudingwa, Banupriya Ravichandrane, Rajasekar Ramadurai, Manasa Rengarajan

**Affiliations:** 1 Anaesthesiology and Critical Care, Jawaharlal Institute of Postgraduate Medical Education and Research, Puducherry, IND; 2 Anaesthesiology and Critical Care, Vinayaka Missions Superspeciality Hospital, Puducherry, IND

**Keywords:** delayed emergence, electrolyte imbalance, hypokalemia, metabolic acidosis, urinary diversion

## Abstract

Delayed recovery from anesthesia may result from unrecognized metabolic and electrolyte abnormalities. We report a case of an 18-year-old female with prior ureterosigmoidostomy who underwent ureteric reimplantation under general anesthesia. She was found to have severe hypokalemia with metabolic acidosis in the perioperative period, which had not been identified preoperatively. Postoperatively, she exhibited neuromuscular weakness and inadequate respiratory effort, requiring elective ventilation. Correction of the underlying abnormalities resulted in rapid recovery and successful extubation. This case highlights the importance of preoperative metabolic evaluation and optimization in patients with urinary diversion to prevent delayed emergence and respiratory compromise.

## Introduction

Urinary diversion procedures involving bowel segments can result in significant metabolic disturbances due to prolonged contact between urine and intestinal mucosa. These include hyperchloremic metabolic acidosis and associated electrolyte abnormalities such as hypokalemia [1,2]. Although often chronic and compensated, these disturbances may become clinically significant in the perioperative period and contribute to complications, including delayed recovery from anesthesia [3]. While metabolic complications of urinary diversion are well-recognized, their role in perioperative delayed recovery remains underreported.

## Case presentation

An 18-year-old female was scheduled for right ureteric reimplantation for ureteric stricture under general anesthesia. She had undergone ureterosigmoidostomy one year earlier for congenital ectopia vesicae with epispadias. Preoperative evaluation revealed no active complaints. Routine preoperative investigations included complete blood count, serum electrolytes, renal function tests, and blood glucose assessment, with borderline low serum potassium (3.2 mEq/L). Relevant laboratory parameters are summarized in Table 1. However, arterial blood gas analysis was not performed, and the patient had discontinued prescribed oral bicarbonate therapy on her own, which was not identified during the preoperative evaluation.

**Table 1:** Arterial blood gas and laboratory parameters at different perioperative time points pCO₂: partial pressure of carbon dioxide; HCO₃⁻: bicarbonate

Parameter	Reference Range	Preoperative	Intraoperative	End of Surgery	Postoperative Day 1
Arterial Blood Gas					
pH	7.35–7.45	-	7.17	7.23	7.40
pCO₂ (mmHg)	35–45	-	43	35	34
HCO₃⁻ (mEq/L)	22–26	-	14.8	17.7	18.5
Potassium (mEq/L)	3.5–5.0	-	2.02	2.32	3.10
Sodium (mEq/L)	135–145	-	144	140	138
Laboratory Investigations					
Hemoglobin (g/dL)	12–15	10.1			9.2
Urea (mg/dL)	15–40	34			31
Creatinine (mg/dL)	0.6–1.2	1.30			1.19
Sodium (mEq/L)	135–145	148			145
Potassium (mEq/L)	3.5–5.0	3.2			3.7
Blood glucose (mg/dL)	70–140	112			148

General anesthesia was induced uneventfully. Neuromuscular blockade was achieved with intravenous atracurium 20 mg (0.5 mg/kg) at induction. During nasogastric tube insertion, the patient developed severe refractory bronchospasm. This was managed with salbutamol nebulization (5 mg), deepening anesthesia with intravenous propofol, intravenous hydrocortisone (100 mg), and intravenous magnesium sulfate (2 g). Following stabilization, surgery proceeded. In view of the intraoperative bronchospasm, intermittent vecuronium 1 mg boluses were used for maintenance in preference to further atracurium supplementation. Given the severe bronchospasm, intraoperative arterial blood gas analysis was performed to assess ventilation, oxygenation, and acid-base status, revealing severe hypokalemia with metabolic acidosis (Table 1). These findings were consistent with metabolic acidosis with associated hypokalemia, with a likely component of respiratory acidosis in the setting of bronchospasm. Potassium chloride (20 mEq) and 7.5% sodium bicarbonate 50 mL were administered based on estimated electrolyte and bicarbonate deficits.

At the end of surgery, following reversal of neuromuscular blockade, spontaneous respiratory efforts were assessed as part of routine emergence evaluation. However, the patient demonstrated inadequate respiratory effort, inability to move limbs, and failure to open eyes. In view of inadequate recovery, extubation was deferred, and elective postoperative ventilation was instituted. Core temperature, blood glucose, and serum calcium levels were normal, and no residual bronchospasm was noted. No significant ECG changes suggestive of hypokalemia, such as U waves or arrhythmias, were observed intraoperatively. Repeat arterial blood gas analysis showed persistent metabolic acidosis with hypokalemia (Table 1).

The patient was transferred to the surgical intensive care unit for elective mechanical ventilation. Correction of electrolyte and acid-base abnormalities was initiated with intravenous potassium chloride infusion (10 mEq/hour) and sodium bicarbonate (50 mEq/hour). After four hours, muscle strength improved, and the patient was successfully extubated. Oral bicarbonate therapy was restarted, and the remainder of the hospital stay was uneventful.

## Discussion

Urinary diversion using bowel segments, particularly ureterosigmoidostomy, is associated with significant metabolic consequences. Absorption of chloride and ammonium ions from urine leads to a chronic acid load, resulting in hyperchloremic metabolic acidosis [1,2]. This condition is reported in up to 10-45% of patients and is typically managed with oral alkalinizing agents such as sodium bicarbonate [1].

**Figure 1:**
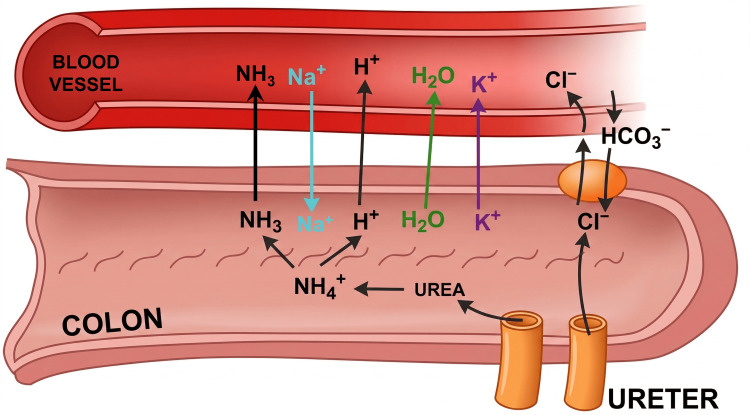
Mechanism of hyperchloremic metabolic acidosis in urinary diversion using colonic segments Figure created using Microsoft PowerPoint (Microsoft Corp., Redmond, WA, US)

When urine is diverted into the colon, prolonged contact between urine and the colonic mucosa results in significant alterations in acid-base physiology. Urinary urea is metabolized by colonic bacteria into ammonium (NH₄⁺), which dissociates into ammonia (NH₃) and hydrogen ions (H⁺). Ammonia readily diffuses across the colonic epithelium into the systemic circulation, while hydrogen ions are concurrently absorbed, contributing to an increased systemic acid load. In parallel, the colonic mucosa actively secretes bicarbonate (HCO₃⁻) into the lumen in exchange for chloride (Cl⁻), resulting in net bicarbonate loss and chloride gain (Figure 1). The cumulative effect of these processes, namely, enhanced acid absorption, bicarbonate depletion, and chloride retention, leads to the development of a chronic normal anion gap (hyperchloremic) metabolic acidosis [1,2].

Electrolyte abnormalities, especially hypokalemia, commonly coexist and may contribute to neuromuscular weakness. In the perioperative setting, these disturbances may be exacerbated by factors such as fasting, fluid shifts, anesthetic agents, and medications [3,4]. The worsening hypokalemia in this patient was likely multifactorial, reflecting chronic potassium depletion related to urinary diversion, interruption of bicarbonate therapy, and possible intracellular potassium shift following beta-agonist administration for bronchospasm [5].

Notably, the initial arterial blood gas analysis demonstrated metabolic acidosis with an inappropriately elevated pCO₂, suggesting a superimposed respiratory acidosis. This was likely secondary to bronchospasm and intraoperative hypoventilation. Subsequent improvement in pCO₂ values reflected recovery of ventilation and partial physiological compensation.

In the present case, discontinuation of bicarbonate therapy and absence of preoperative acid-base evaluation likely contributed to unrecognized metabolic derangement. The administration of beta-agonists for bronchospasm may have aggravated hypokalemia, leading to profound muscle weakness and delayed recovery from anesthesia. Although delayed emergence is multifactorial, electrolyte imbalance and metabolic acidosis were the predominant contributors in this patient. Rapid improvement following correction of electrolyte and acid-base abnormalities further supported these abnormalities as the principal contributors to delayed recovery.

This case highlights that even clinically asymptomatic patients with urinary diversion may harbor significant metabolic derangements, underscoring the need for routine preoperative acid-base evaluation. Preoperative arterial blood gas analysis should be considered in such patients, even in the absence of overt symptoms. Perioperative continuation of alkalinizing therapy and close monitoring of electrolytes are essential to prevent complications.

## Conclusions

Patients with urinary diversion are at risk of metabolic acidosis and electrolyte disturbances that may significantly impact perioperative outcomes. Failure to identify and correct these abnormalities can lead to complications such as delayed recovery and respiratory compromise. Thorough preoperative metabolic assessment, continuation of appropriate medical therapy, and vigilant intraoperative monitoring are crucial in these patients. Increased awareness among anesthesiologists can help optimize perioperative care and improve patient safety.
